# We Need to Improve Prenatal Screening Practices in Primary Obstetric Care: A Representative Data from a Fetal Medicine Unit in Coastal Karnataka

**DOI:** 10.1007/s13224-021-01456-3

**Published:** 2021-03-16

**Authors:** Vidyashree Ganesh Poojari, Sanghamitra Paladugu, Akhila Vasudeva, Anjali Mundkur, Muralidhar V. Pai, Pratap Kumar

**Affiliations:** Department of Obstetrics and Gynecology, Kasturba Medical College, Manipal, Manipal Academy of Higher Education (MAHE), Manipal, Karnataka India

**Keywords:** Primary health care, Primary obstetric care, Prenatal screening, Prenatal diagnosis, Anomaly scan, Mid-trimester targeted scan, Aneuploidy screening, Soft markers, Delayed diagnosis, Fetal medicine

## Abstract

**Background:**

The present observational data from the fetal medicine unit aim to identify gaps in prenatal screening modalities employed in the primary obstetric care population in coastal Karnataka.

**Methods:**

A retrospective observational study of all referrals to Fetal Medicine unit is over 2 years. For each fetal abnormality, we reviewed the literature to note the range of gestational age at which the abnormality should almost always be diagnosed. Taking this as standard, the gestational age at which each of these problems was diagnosed and referred was noted down. They were compared and analysed to understand the efficiency of prenatal screening practices in the referral population. The final perinatal outcome was also noted down in order to assess the impact on perinatal mortality/morbidity.

**Results:**

A total of 277 cases were referred to fetal medicine unit. Two hundred twenty-eight cases (82.31%) were low risk pregnancies. Among 277 cases, 200 (72.2%) had structural abnormalities, 7 (2.5%) chromosomal/ genetic abnormalities, 61 (22.02%) isolated soft markers, and 9 (3.2%) twin-related problems. Detection rate of structural abnormalities was 33% at 14 weeks and 52.22% at 20 weeks, considering those anomalies usually diagnosed by these gestational age windows. The primary reason for delayed diagnosis was non-performance of ultrasound “on time”, rather than missed diagnosis. Fifty-three per cent (106 out of 200) of all the fetal structural abnormalities were diagnosed beyond 20 weeks. Average gestational age at mid-trimester anomaly scan in this group was between 20 and 24 weeks. Sixty-one patients were referred due to isolated soft markers, 30 beyond 20 weeks. Eighty per cent of them did not have any aneuploidy screening in pregnancy.

**Conclusion:**

Practice of fetal medicine hugely depends upon appropriate prenatal screening practices in the referral population. There is an urgent need to bring in standard protocols for Prenatal Screening across all the primary obstetric care providers, both in the public and private sectors. Considering the huge burden of delayed prenatal diagnosis in our country, the proposed revision of MTP bill is a welcome change in fast-growing field of fetal diagnosis and therapy.

## Introduction

Pregnancy outcome heavily depends upon the timely diagnosis and referral from the primary obstetric caregiver. Prenatal detection rate of fetal abnormalities ranges from 17 to 85% [1], depending upon the study settings and the risk profile of study population [2–5]. While managing referrals to fetal medicine unit, we observed severe deficiencies in timely diagnosis at primary care level. We performed a retrospective review of our Fetal Medicine Referrals over the last 2 years, specifically looking for a representative data on prenatal screening practices at primary obstetric care in the referral population.

## Methods

**Fig. 1:**
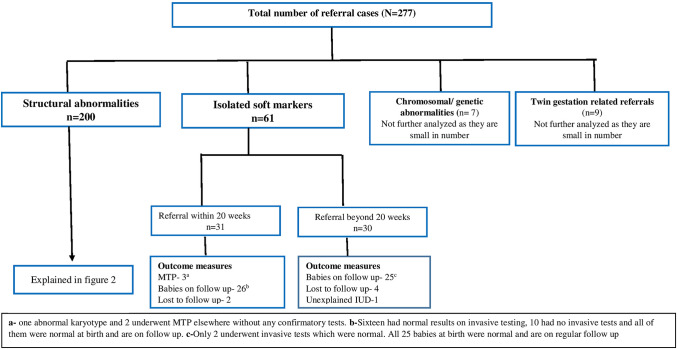
Flow chart illustrating the analysis of 277 cases referred to Fetal Medicine Unit

This is a retrospective observational study on the 2-year referrals to tertiary fetal medicine unit in coastal Karnataka. Study was approved by the Institutional Ethical Committee (600/2017). Each case was reviewed and reasons for referral were broadly categorized as shown in Fig. 1. Pre-existing risk factors were identified. Twenty weeks was taken as an important GA (gestational age) parameter for referral in this study, this being legal upper limit of Medical termination of Pregnancy (MTP) in India. For each fetal structural abnormality, we reviewed the literature to note the GA or a range of GA at which the abnormality should almost always be diagnosed [6]. Taking this as standard, the GA at which each of these structural abnormalities were diagnosed and referred—was noted down from case records. The delay in diagnosis if any, was noted down for each patient. For those with delayed diagnosis, we collected the following information from the case records to know the reason for delay. This included information on number, type, and GA at fetal ultrasounds done in that pregnancy as well as aneuploidy screening at primary care level, if any. Information on their first antenatal visit was not available in many of the case records. Information was gathered on invasive testing and the results. The final perinatal outcome was also noted down in order to assess the impact on perinatal mortality/morbidity. Many patients were referred back to primary care. Follow up information on these pregnancies including neonatal information was collected from the referring doctors.

## Results

A total of 277 cases were referred to fetal medicine unit over a period of 2 years. Mean maternal age at referral was 27.9 years ± 8.2. Out of 277 cases, 170 (61.37%) were primigravida, and 107 (38.6%) were multigravida.

Two hundred twenty-eight cases (82.31%) were not found to have any contributory risk factors for fetal abnormality like age above 35 years, consanguinity, relevant obstetric history, teratogenic drug exposure, infection, or relevant family history.

Among 277 referrals, 200 (72.2%) belonged to the category of structural abnormalities, 61 (22.02%) isolated soft markers, 7 (2.5%) chromosomal/genetic abnormalities, and 9 (3.2%) twin-related problems (Fig. 1). Table 1 shows major system involvements along with their GA at detection/referral.

**Table 1:** Detection of fetal abnormalities according to organ system (*N* = 277)

Organ system	Number of cases *n* (%)	Detected by 14 weeks	Detected before 20 weeks	Detected after 20 weeks
Central nervous system	44 (15.8)	8	23	13
Cardiovascular system	23 (8.3)	1	14	8
Renal	39 (14.07)	0	9	30
Gastrointestinal system	19 (6.85)	0	2	17
Musculoskeletal	17 (6.13)	0	5	12
Face	5 (1.8)	0	3	2
Multiple anomalies	16 (5.78)	2	7	7
Abdominal wall	3 (1.08)	3	0	0
Congenital Diaphragmatic hernia	9 (3.24)	0	1	8
Cystic hygroma	6 (2.16)	5	1	0
Limb body wall defects	5 (1.8)	3	1	1
Hydrops fetalis	3 (1.08)	1	0	2
Arthrogryposis	2 (0.72)	0	0	2
Meckel Gruber syndrome	1 (0.36)	1	0	0
Heterotaxy	2 (0.72)	0	0	2
Placental	4 (1.44)	0	3	1
Lung	2 (0.72)	0	1	1
Chromosomal/genetic abnormalities	7 (2.52)	Not analysed further due to small number		
Soft markers	61 (22.02)	5	25	31

**Fig. 2:**
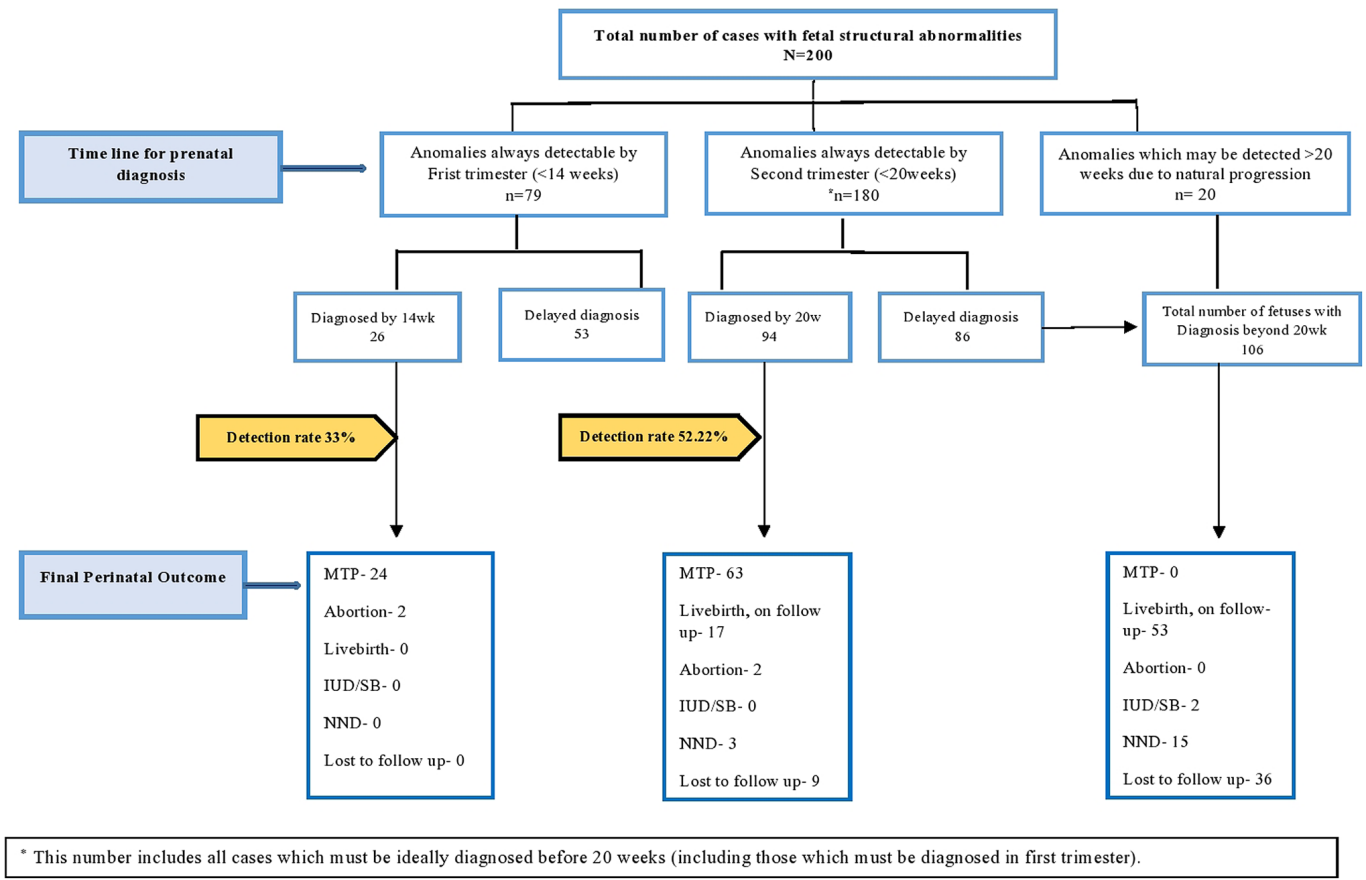
Flow chart illustrating referrals with structural abnormalities in the fetus

Figure 2 shows the timeline of prenatal diagnosis in the referral population. We have represented the two common GA windows at which, most of the fetal abnormalities should almost always be diagnosed [6]. The data on how many anomalies were actually diagnosed in these GA windows are given, along with the data on delay in diagnosis. Perinatal outcomes in each group are shown as—MTP before legal limits of 20 weeks, miscarriage, intrauterine death, stillbirth, neonatal death, live births, and cases lost to follow up.

Out of 200 referrals with structural abnormalities, 79 (39.5%) belonged to the category almost always detectable by 14 weeks GA, out of which 26 were actually diagnosed in that GA (33%). In most cases where the diagnosis was delayed (47 out of 53), the primary reason was non-performance of NT scan in the referral pool rather than “missing” the early diagnosis.

Out of 200 referrals with structural abnormalities, 180 (90%) belonged to the category almost always detectable before 20 weeks (Fig. 2). However, timely diagnosis was achieved only in 94 (52.22%) within this category. It is important to note that—out of 86 cases that were not diagnosed by 20 weeks, diagnosis was achieved by 24 weeks in the majority (70 out of 86). The average delay in diagnosis was 3–4 weeks, beyond 20 weeks of gestation.

There were some fetal abnormalities (20 cases) that are known to be variable in presentation during pregnancy leading to diagnosis in third trimester due to their natural progression (Fig. 2). These were mainly gastrointestinal tract (GIT) abnormalities like bowel obstruction, renal (hydronephrosis), Congenital Diaphragmatic Hernia (CDH) and Central nervous system (CNS) abnormalities like ventriculomegaly.

It is important to note that 53% (106 out of 200) of all the fetal structural abnormalities were diagnosed beyond 20 weeks. Average gestational age at mid-trimester anomaly scan (MTAS) in this group was between 20 and 24 weeks. In our referral population, obstetrician refers to radiologist for the anomaly scan, which is mostly done in the mid-trimester. As per patient’s hospital records, MTAS was done beyond 20 weeks among 45.12% (125 out of 277) of all our patients, which seems to be the reason for delayed referral. Substantial proportions of these delayed anomaly scans were performed between 20 and 24 weeks (104 out of 125, 83.2%).

Among those diagnosed beyond 20 weeks (106 cases), 36 were lost to follow up. We could not gather any information on these pregnancy outcomes; including whether they went ahead with late termination or continuation of pregnancy.

Seven fetuses were diagnosed with chromosomal/genetic abnormalities, following invasive testing performed for appropriate indications. We have not analysed them further as this number is very small to draw any meaningful conclusion on clinical practice at primary obstetric care facilities.

Sixty-one patients were referred due to isolated soft markers, outcome has been presented in Fig. 1. Eighty per cent of them (48) did not have any aneuploidy screening in pregnancy, leaving only age-based a priori risk for further modifications. Couple found it difficult to decide on further options, especially those referred beyond 20 weeks.

Out of 9 twin gestation referrals, 6 were monochorionic diamniotic twins (MCDA), and 3 dichorionic diamniotic twins (DCDA). Main reasons for their referral were twin specific complications such as Twin to Twin Transfusion Syndrome (TTTS), discordant growth or anomaly. We have not analysed them further as their numbers are small precluding any further conclusion from analysis.

## Discussion

Advanced prenatal diagnosis is now available due to the high-end USG machines, 3D/4D multiplanar interpretations, supplemented by rapidly advancing technologies in prenatal genetic diagnosis [7, 8]. Internationally trained accredited foetal medicine specialists are now in India who can even perform advanced therapeutic interventions. However, any beneficial impact on perinatal health indicators would depend upon the prenatal screening practices at primary obstetric care. We attempted to retrospectively analyze the prenatal screening practices, taking a small sample of 2-year referrals to tertiary fetal medicine unit in coastal Karnataka.

Commonest single prenatal screening modality available to general obstetric population is the targeted Mid-Trimester Anomaly Scan (MTAS) ideally performed between 18 and 20 weeks-due to restrictions laid down by the MTP act. However, this essential prenatal screening was not provided to about half of our referral population in a timely fashion. Despite the availability of international and national guidelines on MTAS [9, 10], prenatal screening practices do not seem to be highly effective in India due to heterogeneity in antenatal care delivery as well as lack of awareness. Primary obstetric caregivers, either in the public or private sector, refer the woman to radiologists or less commonly to trained obstetricians for MTAS. There seems to be lack of standardized protocol with respect to GA at referral. There is great heterogeneity in the level of training, experience, expertise, and the protocol of anatomical survey followed by these sonographers.

### Real-Time Efficacy of MTAS in India

In the present study, 106 (53%) out of 200 structural abnormalities and almost 50% of isolated soft markers were diagnosed beyond 20 weeks. It is important to note that only 20 of these 106 belonged to the category—“may be detected in third trimester”—example Congenital Diaphragmatic Hernia (CDH), Congenital Pulmonary Airway Malformation (CPAM), Bowel atresias, etc. The remaining 86 are the structural abnormalities almost always detectable by 20 weeks—for example, anencephaly, holoprosencephaly, common arterial trunk, double outlet right ventricle, Bilateral renal agenesis, cleft lip + palate, limb body wall complex, heterotaxy and multiple anomalies—as shown under various system wise anomalies in Table 1. Most of the fetal abnormalities occur among low-risk population. Upon referral to fetal medicine unit, advanced imaging, multidisciplinary input, cross consultations, invasive diagnostic procedures, and further planning would take another 1–2 weeks. There are several modifiable factors at primary care level—GA at the first antenatal visit, advice on MTAS by the obstetrician, patient compliance, appointments available for MTAS, patient load, overall awareness among doctors/general public. Unfortunately, delayed prenatal diagnosis increases the possibility of late terminations, which in turn invite risks to the mother’s life. As shown in Fig. 1, they also contribute significantly to perinatal morbidity/mortality—including probably preterm delivery, and long-term disabilities/health sequelae in children.

Our data show that delay in performing MTAS is more a problem than a poor sonologic detection rate. Majority (83.2%) of delayed MTAS being done between 20 and 24 weeks in our referral pool, this GA window seems to be preferred by the sonographers for fetal anatomical survey as well as fetal echo. Organ development is near-complete in this GA window. Poor sonological window due to factors like maternal obesity is less troublesome at this gestational age.

Similar reports of delayed prenatal diagnosis have been published from China in 2008 [11]. In their cohort, nearly half had their first antenatal check-up in the latter half of pregnancy and none underwent aneuploidy screening. Chawla et al. [12] in 2012 published a retrospective review of pregnancies diagnosed with foetal anomalies, from District Maternity Hospital in same geographic location of coastal Karnataka. Similar to our study, majority of fetal anomalies were in low-risk obstetric population. Among those who had antenatal check-ups before 20 weeks, only 26.4% had MTAS before 20 weeks. One-third of the affected pregnancies had their MTAS delayed beyond 28 weeks. Eight years later, prenatal screening practices have not improved significantly in this geographical location. Antenatal counselling must include the need for timely MTAS. Chawla et al. [12] observed an overall low rate of anomaly detection, as MTAS missed 44% of anomalies. In our study, information on the first ANC was not available in many records. Although MTAS were done beyond 20 weeks, anomalies were promptly detected in the majority.

In a retrospective study performed in North India in 2015 [6], 209 out of 312 (66%) major structural malformations were detected beyond 20 weeks. Half of them had their anomaly scan only after 20 weeks, and in the other half anomalies were missed during MTAS done before 20 weeks. Like in our series, most of their patients were referred beyond 20 weeks from the primary care level.

### Utilization of the 11–14 weeks’ Scan

Among the fetal anomalies almost always detectable in 11–14 weeks’ scan, only 33% were detected in the study population. Among the “delayed diagnoses” were anencephaly, holoprosencephaly, megacystis, limb body wall complex and multiple malformations. Detection rates are similarly low in earlier publications involving low-risk populations, performed without adherence to a strict protocol at the primary care level [13–15]. Detection rates are higher among high-risk population using systematic protocol [16–18]. It is important to note that a substantial proportion of “non-detection” at 11–14 weeks (45 out of 53 cases, 85%) in our study was due to “non-performance” of 11–14 weeks’ scan, rather than “missing the anomaly”. Hence 11–14 weeks’ scan is not used optimally in our referral population, despite the availability of expertize and good socio-economic indicators in this part of the state [19]. Similarly, Kashyap et al. have reported very low (1.6%) detection rate for the 11–14 weeks’ scan from North India in 2015 [6]. There is an urgent need to increase awareness on the potential benefits of 11–14 weeks’ scans among primary obstetric caregivers.

At the same time, 11–14 weeks’ scan cannot replace MTAS which is an essential screening. Maternal morbidity due to a medically induced abortion at 13 weeks versus that at 19 weeks would not be very different from each other. Therefore, our first aim should be to achieve 100% coverage for an 18-week MTAS. Only later we could aim at achieving a good coverage for 11–14 weeks’ scan. A detailed first-trimester survey should however be offered to high-risk women. A good 11–14 weeks’ scan requires accredited sonographer, currently available only in secondary care level, tertiary institutions, and the corporate sector, reaching only a fraction of our obstetric population.

### Aneuploidy Screening Versus Soft Markers

Similar to the NT scan, aneuploidy screening is not performed in a standardized manner across the country. As in our series, a good number (30 out of 61) are detected to have isolated soft markers in the MTAS performed beyond 20 weeks, without having had any form of aneuploidy screening (80%). Juggling with likelihood ratios based only on age-based a priori risk is inaccurate. This only increases parental anxiety on aneuploidy, not giving them clear-cut options for further testing. Many even requested termination without further testing, due to uncertainties. If only one prenatal screening can be done to all, it would be better to combine quadruple testing along with MTAS at 18 weeks. This strategy gives a close to 80% aneuploidy detection along with a good detection rate of major structural abnormalities [20].

Lastly, psychological impact of prenatal diagnosis must be born in mind [21]. Normal findings in ultrasound (USG) result in joy and psychological well-being. In the absence of appropriate pre-USG counseling like in our country, parents go through much emotional turmoil while receiving the bad news. Although they are committed to pregnancy, they are unable to support a severely disabled child in the absence of social/financial support. These decision dilemmas are further exacerbated due to late diagnosis when the mother has already felt fetal movements and thus emotionally bound to the developing fetus. Recently, many couple have approached the court with a writ petition to allow for late terminations in case of lethal/severe anomalies diagnosed beyond 20 weeks. A huge number of pregnant women are currently undergoing these decision dilemmas—leading to significant morbidity and psychological trauma.

Our data strongly support the need for implementation of proposed amendment to the MTP Act—2020 (Bill No 55 of 2020, The Medical Termination of Pregnancy (Amendment) Bill, 2020 introduced in Lok Sabha). Among those with late detection in our series, a substantial proportion was referred with major fetal abnormalities between 20 and 24 weeks. Using advanced ultrasound technology, many more fetal abnormalities can be diagnosed between 20 and 24 weeks, compared to 18–20 weeks MTAS. Advances in genetic testing like chromosomal microarray and clinical exome sequencing have enabled us to diagnose fetal abnormalities with a dismal prognosis. However, such genetic testing takes a minimum of 2–4 weeks after the detection of structural abnormality in MTAS. Safe abortion methods have substantially reduced the morbidity related to MTP in the mid-trimester.

## Conclusion

There is an urgent need to bring in standard protocols for Prenatal Screening across all the primary obstetric care facilities, both in the public and private sectors. The proposed revision of MTP bill is a welcome change in the country's fast-growing field of fetal diagnosis and therapy.
